# Co-designing strategies to support patient partners during a scoping review and reflections on the process: a commentary

**DOI:** 10.1186/s40900-021-00272-3

**Published:** 2021-05-10

**Authors:** Tamara L. McCarron, Fiona Clement, Jananee Rasiah, Karen Moffat, Tracy Wasylak, Maria Jose Santana

**Affiliations:** 1 The Department Community Health Sciences, Teaching Research and Wellness Building, 3280 Hospital Drive NW, Calgary, Alberta T2N 4N1 Canada; 2 O’Brien Institute for Public Health, Teaching Research and Wellness Building, 3280 Hospital Drive NW, Calgary, Alberta T2N 4N1 Canada; 3 Faculty of Nursing, University of Alberta, 3-141 Edmonton Clinic Health Academy (ECHA), 11405 87 Avenue, Edmonton, AB T6G 1C9 Canada; 4 Patient Partner, Calgary, Canada; 5 Alberta Health Services, 10301 Southport Rd SW, Calgary, AB T2W 1S7 Canada; 6 Faculty of Nursing, University of Calgary, Professional Faculties 2259 2500 University Drive NW, Calgary, AB T2N 1N4 Canada; 7 The Department Psychology, University of Calgary, 2500 University Dr NW, Calgary, AB T2N 1N4 Canada

**Keywords:** Patient and public involvement, Co-production, Patient engagement, Scoping review

## Abstract

**Background:**

Patient partners can be described as individuals who assume roles as active members on research teams, indicative of individuals with greater involvement, increased sharing of power, and increased responsibility than traditionally described by patient participants who are primarily studied. A gap still remains in the understanding of how to engage patients. The objective of this commentary is to describe the involvement of four patient partners who worked with researchers during a scoping review.

**Main body:**

We describe approaches to meaningfully engage patient partners in conducting a scoping review. Patient partners were recruited through existing patient networks. Capacity development in the form of the training was provided to these four patient partners. Engagement strategies were co-designed with them to address potential barriers of involvement and acquiring the necessary skills for the successful completion of this scoping review.

**Conclusion:**

Involving patients partners early in the project established the foundational relationship so patient partners could contribute to their fullest. We witnessed the success of working alongside patient partners as members of the research team with a clear and mutually agreed upon purpose of the engagement in health research activities and how this seemed to contribute to an effective and rewarding experience for both researcher and patient partner.

**Supplementary Information:**

The online version contains supplementary material available at 10.1186/s40900-021-00272-3.

## Plain English summary

Patients have taken on roles as members of research teams, often involved in a wide range of activities, such as members of advisory committees. Despite these important new roles for patients, broadly, researchers are still challenged with identifying opportunities of benefit to all involved. We describe the involvement of four patient partners who worked with researchers to complete a scoping review. We detail the approaches used to support the project and the benefits and challenges we experienced while involving patients in this review. Co-designing engagement approaches assisted in gaining the support and buy-in necessary from patient partners for the successful completion of this scoping review. Involving patients partners early in the project established the foundational relationship for patient partners to contribute to their fullest. We witnessed the success of working alongside patient partners as members of the research team with a clear and mutually agreed upon purpose of the engagement in health research activities and how this seemed to contribute to an effective and rewarding experience for both researchers and patient partners.

## Background

Globally, patient and public involvement (PPI) organizations such as INVOLVE in the United Kingdom [1], Patient-Centered Outcomes Research Institute (PCORI) in the United States [2] and the Canadian Institutes for Health Research (CIHR) Strategy for Patient-Oriented Research (SPOR) in Canada [3] provide infrastructure to support the involvement of patients and their families (PPI) in health research.

Although patient engagement has gained attention as an approach to improve health research and practice, uncertainty still persists about how to involve patients in effective and productive ways in health research [4–6]. This uncertainty stems from the lack of peer reviewed evidence describing the process of patient engagement. Staley argues a gap still remains in understanding how patient engagement impacts research outcomes, and suggests the solution is providing more detailed accounts of patient involvement [7]. This commentary describes the process of patient engagement while conducting a scoping review [8]. The scoping review was undertaken to understand how patients were engaged as partners in health research.

Patient partners are individuals who assume roles on research teams with greater involvement, increased sharing of power, and increased responsibility [9]. CIHR defines ‘patient partner,’ as individuals with lived experience that are involved in and when patients contribute to the research process and research-related activities as equal partners [10]. For instance, patient partners are involved in, supporting grant applications, assisting with participant recruitment, and performing research dissemination activities [11]. This scoping review was conducted by a team of researchers and patient partners. Patient partners were members of the research team, and their role was to help clarify the research question and search terms, lead and conduct the grey literature component of the review and assist with interpreting the results and disseminating the findings.

To address potential barriers such as lack of training for patient partners identified by Bird et al. in a recent scoping review, in which Bird found critical barriers and facilitators to PPI including the lack of training for patient partners [12], we co- designed training activities to support patient partners throughout this project. We describe the process of involvement of four patient partners in this review by characterizing the training provided and co-designed engagement strategies to address potential barriers and facilitators identified as a result of involvement.

### Ethics

Ethics approval was not required because patients involved in this project were not research participants rather, they acted as members of the research team.

### The research team

Two senior faculty members (second and last author), a health authority leader (fifth author), a Post-doctoral Scholar (first author), a PhD Candidate (third author) and four patient partners (one of which is an author), participated in the full project and formed the scoping review team. One partner had to excuse themselves from the project due to family commitments. Three additional students joined the team during the review process to assist in the academic component of the scoping review.

#### Project design

To support our working relationship, we followed the CIHR Strategy for Patient Oriented Research, Guiding Principles [13]. In the following sections, we describe how patients were recruited, how patients were involved, and the engagement strategies used to support the team including the training provided to patient partners during the scoping review.

#### Recruitment

Posters about the patient partner opportunity were distributed among the first authors’ personal networks, and other organizations including the Alberta SPOR Support Unit [14] and Albertans for Health Research [15]. The project description, proposed role, and anticipated time commitment were included in the recruitment poster. Patient Partners were compensated $200 CAD for this project. Interested individuals responded by email providing information about themselves, why they wanted to be involved, and one thing people should know about them. Two presentations were given to highlight the opportunity over the course of a month. See Additional file 1. Five individuals responded to the opportunity. All five individuals were interviewed and later selected. These individuals represented a diverse group in terms of diversity such as sex and ethnicity, and had varying experience working on research projects ranging from no experience to some experience.

#### How were patients involved?

Patients were involved in each phase of the project from refining the research question to dissemination. During the Initial Project Meeting, patient partners and the first author met for three hours to review the overall project, discuss the responsibilities of the patient partners and to establish ground rules to better define how the team would work together. A *Project Development*, a meeting was held with the research team, including our patient partners and key stakeholders such as the Strategic Clinical Networks™ at Alberta Health Services, to refine the research question and search terms used to conduct the scoping review. During *Data Collection*, the patient partners and first and third author modified the Canadian Agency for Drugs and Technologies in Health (CADTH) Grey Matters [16] tool for searching grey literature to identify an exhaustive list of organizations with a focus on patient engagement such as INVOLVE in the United Kingdom and SPOR in Canada. Patient partners participated in *Conducting the Review*, by searching the identified websites for evidence of patients who were engaged as research partners. We utilized the framework by Manafo et al., which described the characteristics of patient as partner by the higher levels of engagement (involve, collaborate, and lead/support) [17] to explore the relationship between study purpose, reported outcomes, and strategies adopted to support partners. During *Data Extraction*, one of the patient partners, who had familiarity conducting scoping reviews, extracted data from all included sources from the grey literature search using a data extraction sheet developed a priori. All data was verified by a second reviewer, the first author. During *Interpretation of Findings*, a second consultation meeting was held to gather input from stakeholders, including the patient partners, and the research team members on preliminary findings to provide context and thoughts to inform the potential implications from the review. Finally, during *Dissemination*, a patient partner participated in authoring this manuscript. See Table 1.

**Table 1:** Stages of Patient Involvement

Stage	Description
Initial project meeting	An initial three-hour meeting was held between the first author, who also acted as the chair, and the patient partners shortly after the recruitment process was completed. This meeting provided an overview of the project and an opportunity for the team to get to know each other and to establish ground rules for working together. Individuals were contacted one week prior by email.
Project development	A project meeting was held with the research team (which included our 5 patient partners, a member of the Alberta SPOR Patient Engagement Platform, and a member of the Strategic Clinical Networks™ at Alberta Health Services. Individuals were asked to assist in the development of the research question and the search terms that would be used to conduct the scoping review. This meeting was 1.5 h in length and occurred early in the project, was chaired by the primary author and established clarity and direction for the project.
Data Collection	We began the data collection phase with a team consisting of academic researchers, graduate students and the patient partners. The patient partners completed the grey literature review. To support the patient partners, the first and third author hosted an initial training session lasting 3 h in length, three weeks after the initial project meeting. This training discussed the purpose of a literature review, the search terms and research question, the steps we would use to conduct the scoping review, the CADTH Grey Matters tool [16], and the framework we would use to select evidence and the roles of each member of the team [17]. The meeting was informally structured to allow the session components to be developed to meet the needs of the team, provided an opportunity for dialogue and an opportunity for the team to get to know each other. Weekly one hour team meetings were established to support the review and to provide additional support and clarity as needed to all members of the research team, including the patient partners. It was discovered early on in the project, that using the Manafo [17] framework was challenging for the students and patient partners. A problem-solving training session was held where a number of examples were chosen and circulated to the team for review. A member of the team was asked to present a record under consideration and to illustrate how it did or did not meet the inclusion criteria. Any additional records that were not clear after this session were flagged and resolved by a third reviewer. The data collection phase occurred over three months.
Data extraction	To facilitate the grey literature data extraction process, one of the patient partners extracted the data using a data extraction sheet developed a priori. This data was later verified by the first author. The other patient partners not participating in data extraction were kept up to date during this phase by using a collaborative communication application called Flock. The data extraction phase occurred over 1 month.
Interpretation of Findings	The research team, including the patient partners, students, a member of the Alberta SPOR Patient Engagement Platform, and a member of the Strategic Clinical Networks™ at Alberta Health Services were invited to a consultation meeting and given a high-level summary of the data extraction sheets. The purpose of this two-hour consultation meeting was to gather input from stakeholders and the research team members on the preliminary findings and to provide context and thoughts to inform the potential implications from the review. Participants were notified of both consultation meetings one week prior by email and this meeting occurred during month seven of the project.
Dissemination	To support the dissemination of these findings, the patient partners were asked if they wished to participate in the publication of the results. The pandemic had reached its first phase of restrictions and lockdowns were ordered. Many individuals declined the offer of writing the manuscript primarily for personal reasons resulting from the pandemic but asked to be kept informed as the project progressed and future opportunities to present our findings including conferences. One patient partner expressed interest in writing and felt this would be an adequate distraction that would keep their mind off what was happening in the world. Writing occurred over 1 month and involved an initial meeting to discuss an outline for the manuscript followed by a back-and-forth process of reviewing and editing until the manuscript was completed.

### Engagement strategies

Some of these strategies were co-designed with the patient partners and others were adopted from previous learnings acquired as a result of working with patient partners [18]. The following section details the different approaches used including establishing the initial relationship and the technology used to support the team throughout the project.

### Developing the relationship

To support engagement, an in-person meeting was held at the start of the project (February 3, 2020). This meeting was held at a local community room that was accessible to patient partners. One partner was unable to travel to the meeting due to illness but did participate via teleconference. This three-hour meeting was chaired by the first author, was a networking opportunity, and served as a way to review the project in greater detail.

Using the four pillars as described by the CIHR Guiding Principles for Patient Engagement Framework as a guide [13], we discussed what meaningful engagement meant to team members and how we would hold each other accountable to upholding the terms of reference we agreed upon. The four pillars of meaningful engagement are: i) inclusiveness (when research includes a diversity of perspectives); ii) support (when adequate support and flexibility are provided); iii) mutual respect (when researchers, practitioners and patients acknowledge and value each other’s expertise); and iv) co-building (patients, researchers and practitioners work together from the beginning to identify problems and gaps, set priorities for research and work together on implementation). Discussion included a wide range of what these pillars meant such as: having flexible meetings, appropriate training and the technology to support their work. The group unanimously agreed to hold each other accountable to the work and developed ground rules to define how we worked together. This initial meeting and the establishment of ground rules helped to address barriers and facilitators to engagement such as time commitment and level of expertise. Time commitment was addressed by being flexible and level of expertise was addressed through the described training sessions. See Additional file 2.

#### Technology

As a result of the Covid-19 pandemic (March 2020), face to face meetings were no longer viable, therefore, in keeping with the commitments to support patient partners with the tools to do the work, an institutional licence for Zoom (https://zoom.us) was provided by the University of Calgary. This enabled the team to continue meeting regularly during this challenging time. Both the Postdoctoral Scholar and PhD Candidate made themselves available to support patient partners with getting software installed, providing some technical assistance with the initial settings including password entry, video settings and answering any questions patient partners had. Using Zoom was a conscious decision as a result of the pandemic and always involved a personal check in with team members and ended with asking if anyone needed assistance. One of the patient partners withdrew at this phase of the project due to family commitments. A communication tool called “Flock” (https://www.flock.com) was introduced to facilitate communication and to assist in building the relationship among team members. This app was very easy to use and was readily adopted by the team members since it was available on handheld devices. Google Docs was used to support data collection and extraction activities.

#### Training

During the initial project meeting, patient partners shared their lack of understanding of undertaking a scoping review. In response to this concern, two weeks following the initial project kick off and team meeting, patient partners and two members of the research team held a three-hour grey literature training session (February 18, 2020). The purpose of this session was an opportunity for the patient partners and research team to further establish their new working relationship, to review and discuss the research question and search terms used in the grey literature search strategy. Given the varying level of experience, this training session was developed together with the patient partners with information shared in a back-and-forth style, including why and how the research is conducted and questions and areas of concern addressed in real time. Most of the questions were related to the level of engagement. For instance, is this study an example of involve or collaborate? How can we differentiate between these two levels? This back and forth dialogue assisted in the patient partners gaining confidence and reinforced their patient partner role as a full member of the research team.

To ensure that all relevant information was captured, the research team modified the CADTH Grey Matters tool for searching grey literature [16] to include an exhaustive list of 58 organizations with a mandate in the area of patient engagement in health research such as INVOLVE in the United Kingdom and SPOR in Canada [8] . After the training session, patient partners worked independently to identify studies, reports, and conference abstracts of relevance to this review.

At the first weekly meeting, it became apparent that confusion with the Patient and Researcher Framework, as depicted by Manafo et al., [17] existed among the patient partners and students. Both were having difficulty determining the differences between the levels of engagement. To help build this knowledge and assist with the remainder of the review, an additional training session was developed and delivered online, with supporting background material to help explain the differences between the levels of engagement defined by Manafo et al. These materials also served as a resource for team members to refer to during their review http://engaging.ucalgaryblogs.ca/research/what-is-patient-and-family-engagement/. To reinforce how to differentiate between the levels of engagement (involve, collaborate and lead/support), individuals would present records they were having difficulty assigning to a level of engagement. Individuals were asked to present the record. These meetings continued until screening was completed. For the grey literature, included records were given to another patient partner for verification. Data extraction was completed by one patient partner and verified by the primary author. Additional one-on-one training was provided on an ad hoc basis to support individuals as needed.

#### Team meetings

Weekly team meetings were held to discuss articles and maintain reliability and quality of the data extraction process. These meetings were held weekly for one hour for the duration of the project (the project lasted for seven months in total). The patient partners, students and researchers were in attendance at the weekly meetings. These meetings also kept the project on track and helped to identify issues or challenges early so they could be addressed. For example, the challenging nature of the spectrum as a lens became clear early on for both the students and patient partners. To address this challenge, an additional training session was developed to help individuals, both patient partners and students, in their understanding of the many different role’s patients could assume in the research cycle and how to differentiate between the levels of engagement as defined by the Patient and Researcher Engagement Spectrum [17]. Patient partners also joined the research team meetings with the students to discuss the scientific literature findings. Patient partners better understood how their work fit into the whole scoping review process. This opportunity had an unintended benefit. Students and patients shared their initial nervousness with meeting each other but at the end of the project both shared their mutual admiration and respect. The students were the most impacted and felt it was an opportunity that exposed them to working with patient partners that they hoped to continue in future research and in their careers. Patient partners shared a newfound respect for the research process and were impressed with the students and their ability to work through the review process. Individual ad-hoc meetings were made available and were most frequent early on in the project as team members were gaining clarity on the review process such as terminology differences between countries (i.e. engagement and involvement).

### Lessons learned

#### Flexibility

As with all projects, when working with patients, flexibility was a key enabler. Illness, a global pandemic, and differing levels of training/experience were all important considerations that needed to be addressed in order for this project to be successful. Early on, when establishing our ground rules for working together, it was important for everyone to commit to attending all of the meetings. Efforts were made to schedule consistent weekly meetings that could be scheduled far enough in advance that if things came up, were able to be rescheduled. Tools like Doodle (https://doodle.com/en/) were used to help the first author find a convenient time to accommodate everyone’s schedules. When the team was impacted by COVID-19, we took time to check in with each other and to make sure we were supporting each other as project timelines were extended and meetings were longer in duration.

#### Motivations

Previous research advanced our understanding of the motivations of patients who engage in healthcare decision-making [19–21]. For example, one of the patient partners was motivated by *Learning New Things* [20]. Understanding how the patient partners were motivated highlighted areas of focus and validated how patient partners made meaning of their involvement. See Table 2 for the complete list of motivations. Using this knowledge, the primary author was able to customize the engagement activities to match the motivations of the patient partners. In the case of this individual (above), after participating in additional training, completed the data extraction, illustrating how delivering a meaningful engagement opportunity, lead to successful completion of research tasks assigned.

**Table 2:** The Motivations of Patients who Engage in Health Research [20, 21]

Motivation	Description of Motivation	Patient Partner Motivation
Self-fulfilment	These individuals are motivated to find purpose; to do something meaningful; and, to establish productive and rewarding connections.	All 5 patient partners shared being motivated to participate in a project that was rewarding and that they could developing connections with other individuals interested in similar things.
Improving Healthcare	These individuals are motivated to make healthcare better; either because of a good/or bad experience themselves or want the healthcare system to perform to the best of its ability.	Two individuals were motivated by the desire to improve the healthcare system and to advocate for other patients, so they did not have to go through what they had experienced.
Compensation	These individuals need to fulfill a financial need (removing barriers to participation); and/or a need to be recognized by others (being paid is an acknowledgement of the patient/family member’s contribution as a partner).	Three individuals were motivated to be seen as an equal player and valued for the perspectives and ideas they brought to the project.
Influence	These individuals need to see (or feel) how they impact decisions; feeling heard; and/or, considered as a “partner.”	Two individuals were motivated to be involved in projects as an equal player and valued for their perspectives.
Learning New Things	These individuals are motivated by the need to be involved in a novel opportunity; curiosity; and self-improvement.	Two individuals were motivated to be involved in projects that provided an opportunity to learn new things.
Conditional	Not like other motivations, these are situation dependent; enhances the choice, to participate or not, by increasing the perceived value to the individual.	Two individuals declared that they chose to participate in the project because the opportunity was flexible
Perks	The symbolic meaning (and prestige) of being a patient advisor and member of the team; Realized when individuals are asked to attend conferences and events on behalf of the organizations and further reinforced when they are supported financially and not out of pocket.	Four individuals indicated they appreciated that there would be opportunities to present the findings from the project at conferences and that their expenses would be covered.

#### Challenges

Additional time was needed, especially at the beginning of the project to provide additional clarity on the project goals, individual roles, training and to establish how we worked together. Luckily, we were able to establish a relationship early on, prior to the pandemic, or our results may have been different. Different levels of training and experience meant that one-on-one meetings with individuals were important and online platforms like Zoom became critical when trying to troubleshoot some of the challenges we faced such as cutting and pasting records into the data collection sheets. Each website was unique, so some patient partners struggled initially with finding the search function and had challenges with wading through all the records as they attempted to assign each to one of the levels of engagement as defined by the Manafo et al., Patient and Researcher Engagement spectrum [17]. This resulted in the primary author reassigning websites to other members of the team in order to meet project timelines.

Patient partners had a different comfort and familiarity with the different technology available such as Microsoft and Apple. These preferences required the first author to provide support for platforms. In addition, these preferences were also present in the data extraction phase, requiring the first author to develop both Microsoft Word and Excel versions of each of the files. In addition, some of the patient partners did not have Microsoft Office so Google Docs was used to support these individuals.

Challenges with the communication app Flock were minor and were due to the different smartphone platforms such as iPhone and Android and were easily addressed by the help files provided by the application developer.

## Conclusion

As a result of this study, we discovered that co-designing engagement strategies to support patient partners assisted in gaining the support and buy-in necessary to successfully support patient involvement. The process described benefits on the research process. Patient involvement in developing the research question and search terms broadened our understanding of patient as partner and as a result helped capture a broader range of studies. Being flexible in our engagement strategies and understanding how individuals were motivated, helped the patient partners gain confidence to be able to lead and conduct the grey literature component of the review. We witnessed how having a clear purpose and being flexible enabled the team to figure things out together was an important strength and likely a key success factor of this project. Customized training also built confidence and the individual capacity of patients with differing levels of training and experience, equipping them with the knowledge to assist in the interpretation of the results and implications of the findings. The process not only contributed to a more relevant review, it also served as an excellent capacity and skill building opportunity for patient partners. Contributing to the awareness and appreciation of not only the research process but also the benefits of involving patients as partners in research. These learnings reinforce the importance of involving patients as partners early in the project to foster and support individuals in building their own confidence and knowledge, so they are able to successfully contribute to their fullest. In summary, the realities of successfully engaging patients in a scoping review require flexibility, training, and specific customized engagement strategies. For example, ad hoc meetings to address challenges and clarify doubts or issues as they arise. Given these findings, we assert the benefits of patient involvement in research balance or exceed any misconceptions of the additional effort and support needed and the mutual benefits to both the patient partners and research team is a worthwhile endeavour for any research team interested in engaging patients in the research process.

## Supplementary Information

**Additional file 1.**

**Additional file 2.** Ground Rules.

## Data Availability

All data generated or analysed during this study are included in this published article and/or its supplementary materials.

## Abbreviations

CADTH: Canadian Agency for Drugs and Technologies in Health
CIHR: Canadian Institutes for Health Research
PPI: Patient and Public Involvement
PCORI: Patient-Centered Outcomes Research Institute
SPOR: Strategy for Patient-Oriented Research
