# Supplementary material for: Co-designing strategies to support patient partners during a scoping review and reflections on the process: a commentary
Source: Res Involv Engagem. 2021 May 10;7:25. doi: 10.1186/s40900-021-00272-3 (PMC8108017; doi:10.1186/s40900-021-00272-3)
Supplement: Supplementary file 1 — Additional file 1. [file 40900_2021_272_MOESM1_ESM.pdf]

Alberta SPOR SUPPORT Unit | Patient Engagement Platform

UNIVERSITY OF CALGARY
O'Brien Institute for Public Health
Health Technology Assessment Unit
Evidence to Inform Policy

LOOKING FOR PARTNERS

We are looking for 3-5 partners to work with us on an exciting new project to develop a patient/family partner strategy that may be used by University of Calgary research teams and others to involve partners in key areas of healthcare decision-making process.

This work will help to inform ways to involve public members in areas of healthcare innovation including health technology assessment, Alberta SPOR and many others.

We are looking for individuals who:

- Are **members of the community** who identify as a patient, family member, caregiver, advisor and who have familiarity with the healthcare system in Alberta;
- Individuals who are **willing to work together**. We plan to co-design this project together.
- Possess a **respectful communication** style;
- Have the **ability to see beyond** their own experience;
- **Comfortable in times of uncertainty** as we explore our way through the project;
- **Ability and willingness** to commit to the entire project; and
- Consider themselves to be **creative problem solvers.**

We are looking for people who have never worked on a research project AND people who have. Even if you have little to no experience being involved in research type projects, we are interested in hearing from you.

The commitment:

- We anticipate that this project will occur over one year, from January 2020 – January 2021.
- We expect the project will involve several phases, with approximately five project milestones including: an evidence-synthesis to ground our work, developing a strategy to involve patients, family members and caregivers in areas of healthcare decision-making, a feedback event to discuss the strategy with key stakeholders, testing the strategy and evaluating the strategy.
- Over the year, we anticipate a commitment of approximately 36 hours (6 meetings lasting 4-6 hours per meeting).
- The meetings and events to support this project will primarily be held at the Teaching, Research and Wellness Building at the University of Calgary, Foothills Hospital site.
- All training will be provided. For example, an understanding of what do we mean by an evidence synthesis.

We will:

- Acknowledge your expertise and perspective as a partner in research;
- Listen to, hear and act on what you tell us;
- Try to schedule meetings at a time that works for you;
- Strive to provide you with information that is clear and understandable;
- Appoint a liaison person from our team who you can turn to with questions and/or ask for clarification of anything that is not clear;
- Be discrete and honour what you have shared with us;
- Cover any parking costs as per the University of Calgary Travel and Expense Handbook- https://www.ucalgary.ca/finance/files/finance/travelexpensereimbursementhandbook.pdf;
- Compensate you for your time and expertise:
    - For each project milestone you participate in, you will be compensated $200 (including preparation time); and,
    - You have the right to decline the compensation offered without it impacting your ability to participate.

If you are interested in participating in this opportunity, please include a little bit of information about yourself, why you want to work on this project and one thing everyone should know about you? You can send your response and indicate your interest by emailing Tamara L. McCarron at tlmccarr@ucalgary.ca by December 20, 2019.
