# Supplementary material for: Co-designing strategies to support patient partners during a scoping review and reflections on the process: a commentary
Source: Res Involv Engagem. 2021 May 10;7:25. doi: 10.1186/s40900-021-00272-3 (PMC8108017; doi:10.1186/s40900-021-00272-3)
Supplement: Supplementary file 2 — Additional file 2. Ground Rules. [file 40900_2021_272_MOESM2_ESM.docx]

Appendix 2: Ground Rules

Using the CIHR Guiding Principles for Patient Engagement (13), the patient partners and first author developed the following document to help define how they would work together.

**RESPECT**

- We are all responsible for ensuring we start and end on time
- If you are unable to participate, please let the research lead or one of your teammates know and make arrangements to get caught up
- Minimize distractions, including cell phones and spamming in the Flock chat
- Work at providing a space for everyone to share what they would like to say
- Strive to understand first, then seek to be understood

**TRUST**

- Everything discussed while in class or in our groups stays there, unless everyone is in agreement it can be shared
- Challenge yourself, be open to new ideas and perspectives
- Rely on each other

**COMMUNICATION**

- Support productive and respectful conversations
- Establish a common language that works for all team members, so everyone feels included

**LEARNING**

- Come prepared to class and meetings, ready to work
- Help each other to understand the ideas/concepts we discuss

**HEALTH**

- We support our physical health by breaking regularly
